# Physiologically stable F127-GO supramolecular hydrogel with sustained drug release characteristic for chemotherapy and photothermal therapy

**DOI:** 10.1039/c7ra12099k

**Published:** 2018-01-05

**Authors:** Bingxia Li, Luna Zhang, Zichen Zhang, Ruoqing Gao, Hongmei Li, Zhipeng Dong, Qiyan Wang, Qingfa Zhou, Yue Wang

**Affiliations:** Key Laboratory of Biomedical Functional Materials, School of Sciences, China Pharmaceutical University Nanjing 211198 Jiangsu Province China zwy_1115@126.com; State Key Laboratory of Coordination Chemistry, Nanjing University Nanjing 210093 Jiangsu Province China

## Abstract

A synthetic method for preparing a Pluronic F127 (F127)-stabilized graphene (GO) supramolecular hydrogel as a safe nanovehicle for combination treatment has been studied. Doxorubicin (DOX) as a model drug is non-covalently bound on the great surface area of GO due to strong π–π interaction, hydrophobic interaction, and the strongest hydrogen bonding. *In vitro* drug release experiments revealed that this F127-stabilized GO supramolecular hydrogel has a sustained drug release characteristic. Furthermore, the supramolecular hydrogel showed better *in vitro* antitumor ability under NIR (near infrared) laser irradiation because of the excellent photothermal effect of GO. Moreover, we evaluated its antitumor ability *in vivo* and the results show that the hydrogel system can also markedly inhibit the growth of a tumor when administered individually, especially under laser irradiation. All these findings make the supramolecular hydrogel system promising for combination therapy with good bioavailability and minimal side effects.

## Abstract 2

The F127-GO-DOX supramolecular hydrogel system with sustained drug release characteristic for chemotherapy and photothermal therapy.

## Graphical abstract

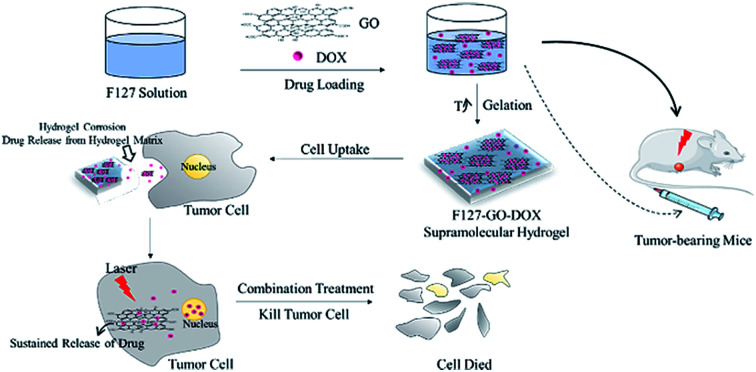

## Introduction

Cancer is a disease involving dynamic changes in the genome and composed aberrant pathway network regulation, which makes curing cancer a formidable challenge.^1^ As a result, various basic research methods and effective treatments have been implemented for cancer therapy, such as chemotherapy, radiation, and gene therapy.^2^ However, the complexity of ontogenesis restricts the application of a single mechanism approach, resulting in lower efficiency.^3^ Combined pharmacotherapy or combinations of chemotherapeutics with other treatment modalities have been proven to be highly effective due to their ability to impact multiple disease pathways *via* different mechanisms.^4^ Photothermal therapy (PTT) is one promising treatment which is based on a photosensitive material such as graphene, which can convert infrared to heat energy and then damage tumor cells.^5,6^ Therefore, the combination of chemotherapeutics with PTT has been regarded as a potent strategy for cancer treatment.

It was also reported that to be a successful combination therapy, the efficacy could be largely improved by the use of a drug carrier as a fundamental tool.^7,8^ Many innovative drug delivery systems such as nanoparticles, carbon nanotubes, liposomes, and micelles have been explored in the biomedical field.^9–12^ Although these current nanovehicles can, to a certain extent, prolong the internal circulation time and improve the targeting of a drug to increase the cellular effective drug concentration, the relatively low drug loading capacity and potential cytotoxicity still hinder their further clinical applications.^13^ Therefore, it is still a challenge to seek for new drug delivery systems with high drug loading efficiency, excellent biocompatibility and low cytotoxicity when applied in delivering antitumor drugs.

In the process of finding new functional materials to apply in the biomedical field, graphene, a two-dimensional nanomaterial, has been widely investigated for its prominent physical properties in many fields.^14^ Moreover, its unique 2D structure and high surface area have attracted much attention in drug delivery.^15^ However, its poor water solubility has limited its further application.^16^ Graphene oxide (GO) bearing functional groups (hydroxyl, carboxyl and epoxide groups) has better water solubility than graphene and can easily be loaded with diverse types of drug molecules through π–π stacking, hydrophobic interaction or hydrogen bonds.^17,18^ It is worth mentioning that GO is an excellent photosensitive material widely used for PTT, which can convert NIR to heat energy efficiently with subsequent damage to tumor cells by significantly increasing the local temperature of the tumor tissue.^19,20^ But the agglomeration or precipitation of GO in electrolyte solutions such as buffered saline seriously restricted its development which needs to be addressed for drug delivery.^21^

To resolve that problem, a Pluronic F127 (F127)-stabilized GO supramolecular hydrogel has demonstrated that it can be physiologically stable in suspension.^22^ Thus GO supramolecular hydrogel derivatives are going to be considered as suitable nanocarriers in drug combination applications. F127, an FDA-approved biocompatible Pluronic polymer, contains two hydrophilic PEO blocks and a hydrophobic polypropylene oxide (PPO) between those two PEO blocks.^23^ As a kind of temperature-dependent phase inversion gel, it will become gel-like from the solution state when the environmental temperature is higher than its gelling-temperature, and this process is reversible.^24^ So in storage conditions it is a free-flowing liquid, but it can fill tissue after injection into a tumor because the phase transition occurs rapidly and the semi-solid state gel is formed at the injection site. In addition to the above, the large drug loading, which can even reach 100%, is another advantage of F127 gel as a drug carrier.^22^ The following drug release process is dependent on the corrosion of the gel, which due to the water molecules entering into the gel network structure and reducing the gel concentration transforms the gel to the solution state even at the gelling-temperature.^25,26^ The release behavior of the drug loaded in the gel can be tuned with the corrosion process. Thus, besides the advantages of biocompatibility, convenient preparation, and high drug loading efficiency, the gel drug delivery system also has a sustained-release property which can delay drug release, making it a good and widely used drug carrier.

In this work, we prepared a supramolecular hydrogel drug delivery system with a sustained drug release characteristic that was composed of F127-stabilized graphene oxide (F127-GO), which could be applied for the combination treatment of chemotherapy and photothermal therapy. The introduction of F127 endows the F127-GO with excellent stability in physiological solutions and low cytotoxicity, as well as weak immunogenicity properties. The cargo release kinetics of F127-GO were studied using doxorubicin hydrochloride (DOX) as a model drug. We also demonstrated that the drug loaded-F127-GO supramolecular hydrogel could inhibit cancer cells efficiently through the combination therapy *in vitro* and *in vivo*. The hydrogel solution can quickly fill around cells or in tumor tissue after injection because of the rapid phase transition at the injection site. Then the hydrogel erosion results in drug release from the hydrogel matrix. Thus the free drug molecules will enter cells through endocytosis together with drug-loading GO, which performs slow drug release and has a photothermal effect in tumor cells, to achieve efficient treatment cooperatively. Thus, the F127-GO supramolecular hydrogel might be a promising controlled platform for combination therapy for cancer.

## Experiment section

### Materials

Graphite powder was purchased from Shanghai Colloid Chemical Plant Co., Ltd. Doxorubicin hydrochloride (DOX·HCl) and Pluronic F127 (F127) were purchased from Dalian Meilun Biology Technology Co., Ltd. Sulfuric acid (H_2_SO_4_), phosphoric acid (H_3_PO_4_), potassium hypermanganate (KMnO_4_), hydrogen peroxide (H_2_O_2_), hydrochloric acid (HCl), and sodium hydroxide (NaOH) were purchased from Nanjing Chemical Reagent Co., Ltd. All reagents were of analytical grade and used as received unless mentioned.

### Characterization

Ultraviolet-visible spectra were collected using a LAMBDA-35 spectrometer. Infrared spectra (4000–400 cm^−1^) were recorded on Bruker Fourier transform infrared (FTIR) in KBr pellets. The X-ray powder diffraction patterns were recorded on an X'Pert diffractometer (PANalytical B.V., Almelo, The Netherlands) with CuKα radiation. Transmission electron microscopy (TEM) was performed on a JEOL-2100 with an accelerating voltage of 200 kV. TEM samples were prepared by drop-casting dispersion onto copper grids covered by carbon film. The scanning electron microscopy (SEM) was performed on a JEOL-S-3400 N II. The atomic force microscope (AFM) images were acquired using an SPM9700. The NIR laser was 808 nm (PSU-III-LRD, CNI Optoelectronics Tech. Co. Ltd, China).

### Exploration of the gelling-temperature to determine the gelling-concentration of F127

The initial concentrations of F127 were determined for 15% wt, 17% wt, 20% wt, 25% wt according to literature searches.^27,28^ Then solutions of different concentrations were prepared by mixing certain amounts of F127 and distilled water. To improve the temperature of each sample, once the sample had changed to a gelling state from solution, the phase inversion temperature was recorded rapidly to choose the most suitable concentration for the following experimental study.

### Preparation of graphene oxide (GO) nanosheet

GO was synthesized *via* a previous method.^29^ In a typical procedure, 1.0 g of graphite powder was put into a flask that was placed in an ice bath. Then, 150 mL of mixed acid (135 mL concentrated sulfuric acid, 15 mL of phosphoric acid) was added to the mixture below 5 °C. Subsequently, 6 g of KMnO_4_ was added to the reaction system stepwise over 1 h below 15 °C. Then, the temperature was raised to 50 °C and the mixture was stirred for 12 h. 200 mL of iced distilled water was slowly added into the solution. Finally, H_2_O_2_ (30%) was poured into the reaction system until no bubbles were observed, resulting in the formation of a bright yellow suspension. After that, graphite oxide was washed with distilled water, HCl (1 M) and distilled water three times, respectively. Finally the product was dried to a brown solid. 100 mg of dry graphite oxide was dissolved in 40 mL of NaOH (0.01 M) and exfoliated by ultrasonication for 4 h. Then, the mixture was dialyzed in distilled water for two days and dried for further use.

### Preparation of F127-GO

Triblock copolymer F127 (1.025 g) was added into the GO dispersion (5 mL) and then the mixture needed to swell at 4 °C for 24 h to obtain a stable F127-GO dispersion.

### Loading of doxorubicin hydrochloride

5 mg of DOX was added into a stable F127-GO dispersion (5 mL) and then the mixture was stirred overnight in the dark. The final drug loaded F127-GO dispersion was brown-colored and stable at room temperature.

### *In vitro* DOX release

The DOX-loaded hydrogel samples were *in situ* prepared in the dialysis chamber, and then dialyzed in 60 mL of phosphate buffer solution (PBS, pH = 7.4, 5.5). The release experiment was conducted in an incubator that was shaken at 50 rpm and kept at a constant temperature of 37 °C. At predetermined time internals, 1 mL of release solution was withdrawn from each sample and fresh PBS of equal volume (1 mL) was added. The amount of DOX released from the hydrogel samples was determined by UV spectroscopy using a standard DOX concentration curve at the wavelength of 492 nm, and the cumulative released amount was calculated.

### Cell culture

A549 (lung adenocarcinoma cell lines) were provided by KeyGEN Biotech and maintained in Dulbecco's Modified Eagle's Medium (DMEM) containing 10% fetal bovine serum, 100 units per mL penicillin, and 100 mg mL^−1^ streptomycin in 37 °C, 5% CO_2_.

### Photothermal treatment and cell viability assay

The cytotoxicity *in vitro* was measured by using an MTT assay against A549 cells. In a typical procedure, cells were initially seeded into a 96-well cell culture plate at 1 × 10^4^ per well and then incubated for 24 h at 37 °C under 5% CO_2_. DMEM solutions of F127-GO, F127-GO-DOX and F127-DOX were added under the same conditions. After incubation for 24 h, the cells were irradiated with the 808 nm NIR laser at a power density of 0.5 W cm^−2^ for 10 min. (The laser beam covers the whole well.) Each sample has a set control group. The cells were further incubated for 48 h at 37 °C under 5% CO_2_. The cells were washed three times with 0.2 mL PBS to remove the unbound NPs. Subsequently, 0.2 mL of DMEM and 25 μL of MTT (5 mg mL^−1^) were added to each well and incubated for an additional 4 h at 37 °C under 5% CO_2_. Then the medium solution was replaced by 0.15 mL of DMSO solution. After 10 min, the optical density at 490 nm (absorption value) of each well was measured on a microplate reader. DOX was used as a positive control for the MTT assay.

### *In vivo* tumor inhibition

All animal procedures were performed in accordance with the Guidelines for Care and Use of Laboratory Animals of China Pharmaceutical University and approved by the Animal Ethics Committee of China Pharmaceutical University, Jiangsu, China. To evaluate the curative effects of the F127-GO-DOX hydrogel drug delivery system *in vivo*, twenty-one mice with tumor models were employed, which were divided into seven groups. Five of the groups were intratumorally treated with saline (100 μL), DOX (100 μL, 1 mg mL^−1^ in saline), F127-GO-DOX (100 μL, 1 mg mL^−1^ in saline), F127-DOX (100 μL, 1 mg mL^−1^ in saline), or F127-GO (100 μL, 1 mg mL^−1^ in saline) per mouse, respectively, for investigation without NIR laser irradiation. The other two groups were intratumorally treated with F127-GO-DOX (100 μL, 1 mg mL^−1^ in saline) or F127-GO (100 μL, 1 mg mL^−1^ in saline) per mouse, respectively, to investigate the photothermal effect under NIR laser irradiation. The mice treated with saline were used as the control group. When saline and samples were injected, the tumor sizes and mouse weights were measured periodically. Seven days later, the groups for investigating the photothermal effect were irradiated by the 808 nm laser at a power density of 0.5 W cm^−2^ for 10 min per mouse. Finally the treated mice from all groups were sacrificed, and the tumor tissues were removed from the bodies for weighing.

## Results and discussion

**Scheme 1:**
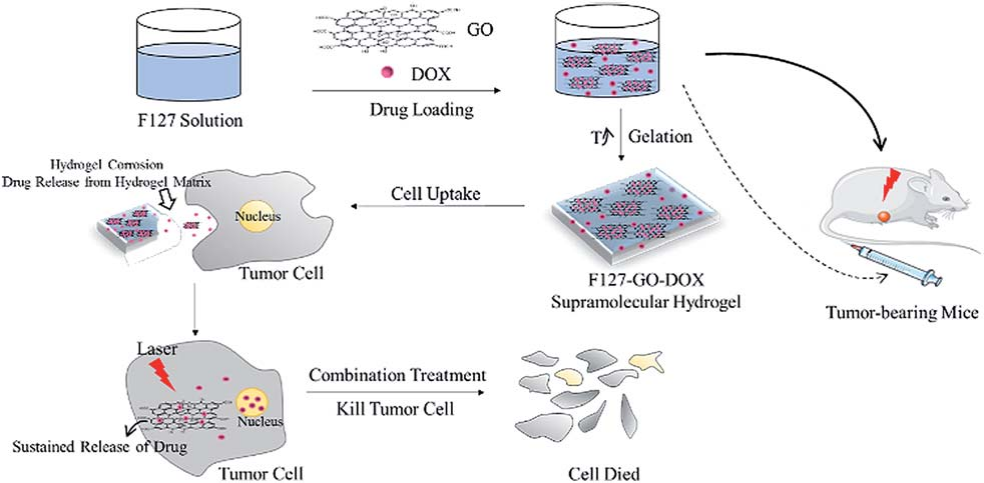
Synthesis and mechanism of F127-GO-DOX supramolecular hydrogel system.

The synthesis of F127-GO supramolecular hydrogel with physiological stability for delivering DOX is shown in Scheme 1. GO nanosheets were extracted from graphite using a modified Hummers' method. Then the F127 were used to form an F127-GO supramolecular hydrogel. The concentration of the F127 was 17% wt and the critical temperature of the final hydrogel system was 28 °C. The corresponding critical temperatures of different concentrations of F127 are shown in Table 1.

**Table 1:** The phase inversion temperature of F127 at different concentrations

wt%	15%	17%	20%	25%
*T*/°C	—	28	23	20

**Fig. 1:**
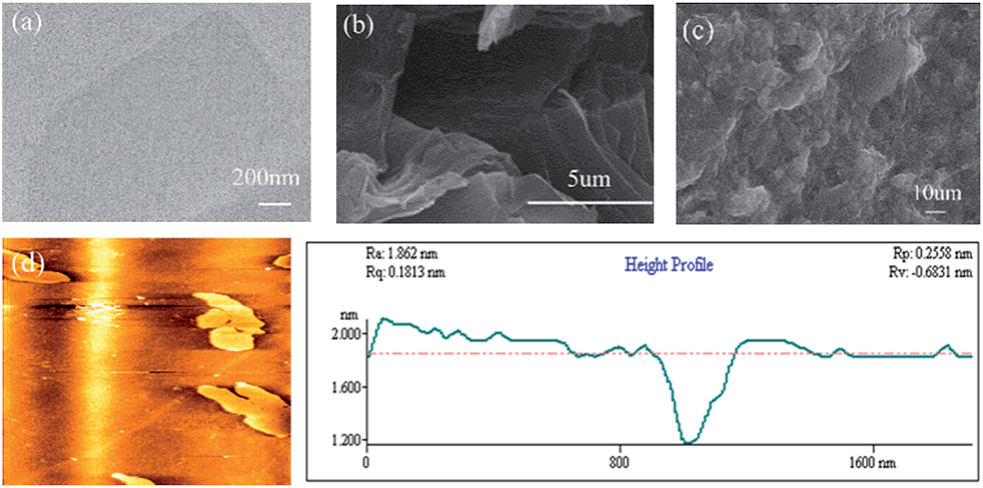
(a) TEM image of GO sheets. (b) SEM image of GO sheets. (c) SEM image of F127-GO. (d) AFM image of GO.

The morphology and structure of the GO and resulting F127-GO supramolecular hydrogel were directly characterized by TEM, SEM and AFM. The ultrafine and single morphology of GO can be observed clearly in Fig. 1(a) and the average thickness of the GO was less than 0.8 nm, as reflected in Fig. 1(d), which proved that GO formed in a single layer through ultrasonication. Compared to the well-dispersed flakes of GO in the low-magnification SEM image in Fig. 1(b), the surface of the F127-GO supramolecular hydrogel (Fig. 1(c)) was very wrinkled and covered with F127 polymer.

**Fig. 2:**
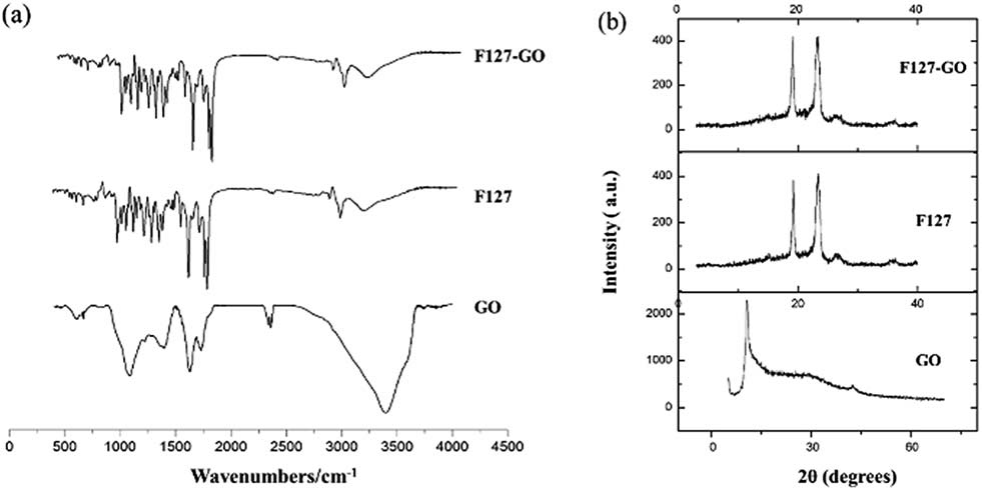
FTIR spectra (a) and XRD spectrum (b) of F127-GO, F127 and GO.

The FTIR spectrum of the F127-GO supramolecular hydrogel is given in Fig. 2(a). Unlike graphite, GO nanosheets had peaks at 1719 cm^−1^ and 1580 cm^−1^, which represent the stretching vibration band of C=O, C–C, and a strong absorption band at 3403 cm^−1^, corresponding to the H–O stretching vibration in GO. When F127 covered the GO, the amount of F127 was far larger than that of GO, so that the nano-sized GO was almost covered by F127. The FTIR spectrum of F127-GO was similar to that of F127, while the characteristic peaks of GO disappeared.

Typical XRD patterns of GO, F127 and F127-GO are depicted in Fig. 2(b). GO showed a strong characteristic diffraction peak at 2*θ* = 10.30°, illustrating the transformation from graphite to GO. The pattern of F127-GO was similar to that of F127 and the characteristic peaks of GO disappeared, results which coincided with those of the FTIR spectra.

In this work, a kind of DNA topoisomerase II inhibitor, DOX, was loaded on the F127-GO supramolecular hydrogel.^30^ A tunable profile of the drug loading and release behavior by the hydrogel was demonstrated. For drug loading, the final concentration of DOX was 1 mg mL^−1^. Due to the great surface area of GO yielding to strong hydrophobic interaction with aromatic compounds, the majority of the drug molecules were loaded on the GO while the remainder were dissociated in the hydrogel matrix. Once the hydrogel formed, the rest of the free drug molecules in the F127-GO dispersion were totally encapsulated into the hydrogel matrix to achieve a nearly 100% loading percentage. Such a high drug loading efficiency was far beyond that of common drug carriers, such as liposome and polymer vesicles, confirming that F127-GO hydrogel is a material with higher drug loading than other drug carriers for drug delivery.

**Fig. 3:**
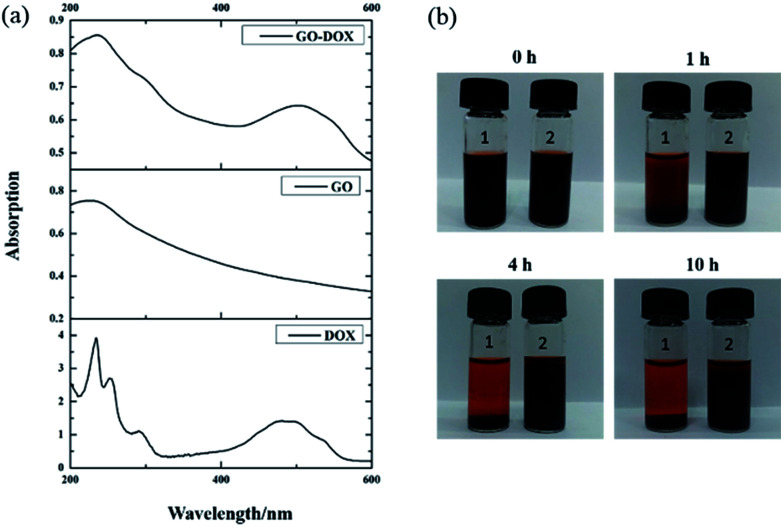
The UV-vis spectra (a) of DOX, GO, and GO-DOX and the digital photos (b) for stability investigation of F127-GO-DOX hydrogel (sample 2) with GO-DOX (sample 1) solution as a control at different times.

After drug loading, to confirm that DOX was indeed bound on the surface of GO, the UV-vis spectra of DOX, GO, and GO-DOX are shown in Fig. 3(a). The UV-vis spectra of DOX before the loading experiment display the characteristic absorption peaks at 233 and 480 nm. After DOX loading onto the GO surface, the characteristic absorption peaks at 233 nm and 480 nm of GO-DOX are broader and lower than those of free DOX. This phenomenon was caused by the π–π stacking interaction, hydrophobic effects and hydrogen bonding between DOX molecules and the GO surface, as reported. The π–π stacking interaction and hydrophobic effects are mainly contributed by the quinone moieties of DOX. Meanwhile the –OH and –NH_2_ groups in the DOX molecules form hydrogen bonds with the –OH and –COOH groups of GO. The above results indicated the effective loading of DOX onto the GO.

We also investigated the stability of the F127-GO-DOX hydrogel system owing to the stabilizing role of F127 after drug loading. As shown in Fig. 3(b), within the first 1 hour, the GO-DOX solution already showed serious agglomeration or precipitation, and the solid was almost completely precipitated in 4 h. The F127-GO-DOX was still stable as a suspension because in the F127-GO-DOX dispersion, the hydrophobic PPO segments of F127 bind to the relatively hydrophobic area of the GO surface, and the hydrophilic PEO chains extend into water to stabilize the GO. These phenomena showed that the addition of F127 could stabilize the hydrogel system without obvious agglomeration or precipitation.

**Fig. 4:**
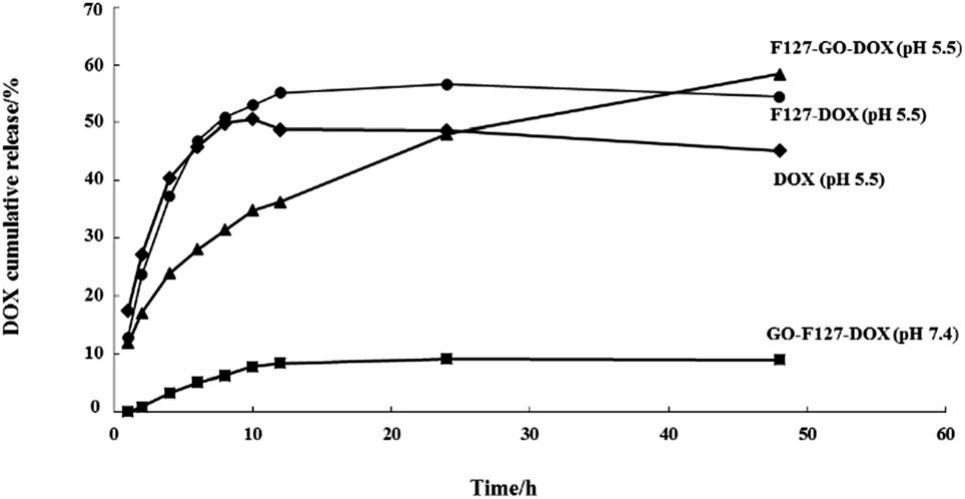
The release percentage curves of DOX from the F127-GO-DOX hydrogel (▲, pH 5.5; ■, pH 7.4), F127-DOX hydrogel (●), and DOX solution (◆).

To further understand the drug release behavior of the F127-GO-DOX hydrogel, we incubated different samples of F127-GO-DOX, F127-DOX, and single DOX solution in phosphate buffers as a pH 5.5 analogue of physiological conditions. And we also investigated the differences in release in a neutral environment. The released DOX was measured by UV-vis spectroscopy of the dialysis solution from different hydrogel samples with time dependence. The release curves are shown in Fig. 4.

At the beginning of the release process, all the different samples in each dialysis chamber gradually dissociated so that the DOX molecules in all the samples were released quickly. But the cumulative release of F127-DOX hydrogel is higher than that of single DOX solution, which is about 55%, illustrating that the hydrogel system could improve the total amount of drug release. When the GO was added into the hydrogel system, due to the strong non-covalent bonding between GO and DOX, the release process of the drug was obviously delayed, as indicated by the 35% release percentage of the F127-GO-DOX hydrogel in the first 12 h. Thus, in this stage, the F127-GO-DOX hydrogel system showed a slower release process than F127-DOX, in which the drug molecules might be released directly from the hydrogel matrix due to its erosion. After 12 h of release, there was some reddish powder-like solid left in the dialysis chamber of the F127-GO-DOX hydrogel, while there was almost nothing left for the F127-DOX hydrogel and very little further release from the F127-DOX hydrogel was observed. Moreover, the DOX molecules were continuously and slowly released from the F127-GO-DOX hydrogel, and release finally reached 60%. We inferred that in the second stage, the DOX drug molecules that were loaded on the GO surface began to be released into the medium because the acid conditions destroyed the interactions between DOX and the GO surface, resulting in an inefficient release under neutral conditions. The contrast in release curves at pH 5.5 and pH 7.4 of the F127-GO-DOX hydrogel also confirmed the above conclusion. In addition, the total amount of the F127-GO-DOX hydrogel released was higher than for the F127-DOX hydrogel, and its release process was much longer. Taken together, all these results demonstrate that the F127-GO-DOX hydrogel system had a release behavior with longer and more sustained properties.

**Fig. 5:**
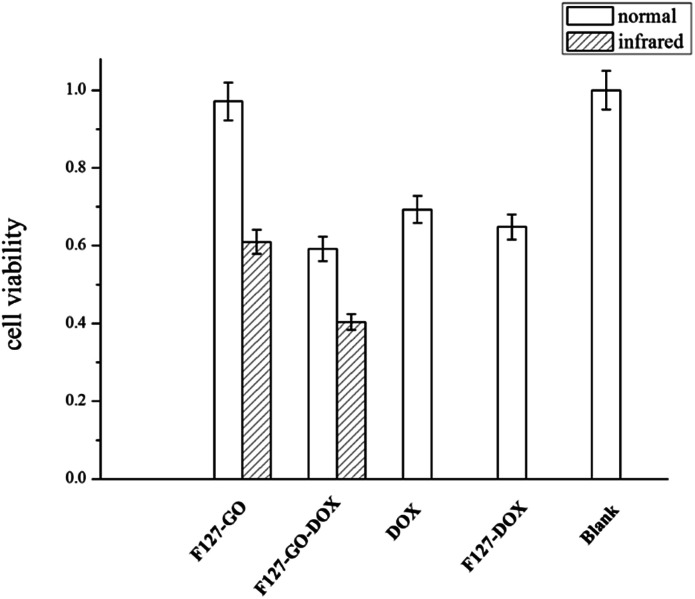
Cell viability of different hydrogel samples against A549 cells under normal and infrared conditions (0.5 W cm^−2^ for 10 min) in 48 h.

For biomedical applications, safety and toxicity are major concerns in using a GO-based drug delivery for cancer therapy.^31^ The *in vitro* cytotoxic activity of the as-prepared F127-GO was investigated on A549 cells by a standard MTT assay. As shown in Fig. 5, the cell viability was always above 90% at a certain concentration, indicating that the F127-GO nanocarrier itself was not cytotoxic and was quite biocompatible, and so suitable for drug delivery.

We then investigated the cytotoxicity of the drug loaded samples of F127-GO-DOX, with DOX as a control. The cell inhibition ratio of F127-GO-DOX, F127-DOX and single DOX were about 41%, 35%, 31%, respectively. It was not surprising that the cell inhibitory capacity of F127-GO-DOX was higher than that of single DOX, which was consistent with their drug release capacity.

Next we gave NIR laser irradiation to the groups of F127-GO and F127-GO-DOX to evaluate the photothermal effect of GO. After laser exposure, both groups showed a significantly improved cell inhibition rate compared to the non-infrared groups. The cell inhibition rate of F127-GO-DOX after laser exposure was increased to about 60% from 41% because of the synergistic effects of chemotherapy and photothermal therapy, and even the nondrug loaded carrier could reach about 40%. Based on these findings, we concluded that the F127-GO-DOX hydrogel drug delivery system had synergistic effects under laser irradiation. These two therapies, chemotherapy achieved by DOX and PTT achieved by GO, showed a much better anticancer effect than expected when used cooperatively. The agreeable effects proved that F127-GO was a potentially outstanding drug combination carrier for a laser-triggered PTT effect along with the chemotherapy under laser irradiation.

**Fig. 6:**
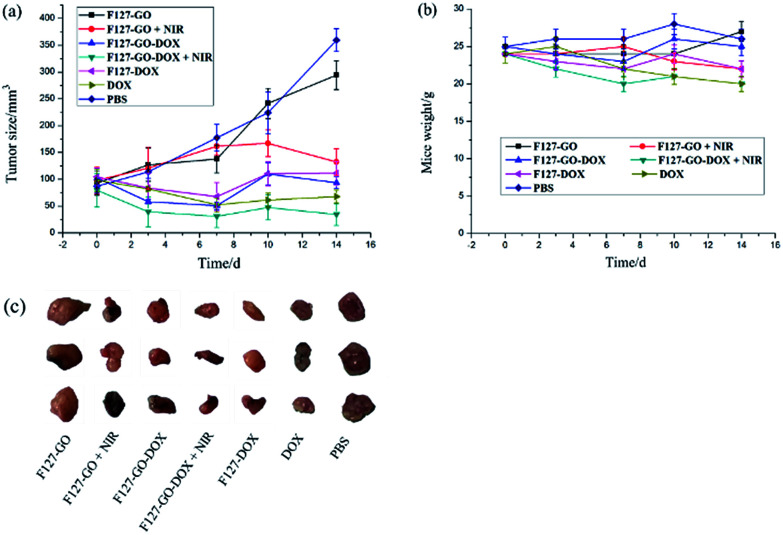
(a) Real-time observation of tumor sizes *in vivo* from the different treatment groups. Error bars represent means ± SD. (b) Real-time weight analysis of mice after each treatment. Error bars represent means ± SD. (c) Tumor tissue from the different treatment groups after 14 days.

Encouraged by the excellent *in vitro* antitumor activity, we then chose A549 cells to establish a subcutaneously implanted tumor model on nude mice. When the tumor volume reached about 60–100 mm^3^, mice were used for the following experiments. On this basis, we investigated the preliminary tumor inhibition effect of F127-GO-DOX. Tumor models in nude mice were established using the method in the Methods section. We periodically measured the tumor sizes after intratumoral injection of different samples. The mice treated with saline were used as the control group. We measured the tumor sizes periodically in order to investigate the tumor growth after administration. Before PTT, Fig. 6(a) showed that *in vivo* growth of the tumor was inhibited to varying degrees when treated with samples of DOX, F127-DOX and F127-GO-DOX, and the group with F127-GO showed no obvious inhibition effect. Moreover, the F127-GO-DOX group had better curative effects than the pure DOX and F127-DOX groups studied over the two-week period. After treatment with F127-GO and F127-GO-DOX for 7 days, we chose two groups to be given 808 nm NIR laser (0.5 W cm^−2^) irradiation to each mouse for 10 min. The other two groups treated with F127-GO and F127-GO-DOX but without NIR were tested for comparison. After photoirradiation, at day 10 and 14, black scars in the tumor tissues were observed in the mice from the groups of F127-GO and F127-GO-DOX with NIR treatment, and no obvious tumor progression was observed. Importantly, the tumor reduction was enhanced because of the photothermal therapy compared to the groups without NIR laser irradiation, and even the F127-GO group obtained a good treatment effect after PTT. As expected, F127-GO-DOX can effectively suppress tumor growth and the curative effect would be improved under photoirradiation. The tumor tissues separated from different treatment groups after 14 days are shown in Fig. 6(c).

We also measured the changes in mouse weight during the treatment process, and the initial weights of nude mice were recorded to be approximately 25.0 ± 0.21 g. As shown in Fig. 6(b), the weights of all seven groups of mice changed to different degrees with 27.3, 22.1, 24.8, 20.3, 22.0, 21.9 and 25.7 g on average for saline, DOX, F127-DOX, F127-GO-DOX (with/without NIR) and F127-GO (with/without NIR) groups at the end of treatment, respectively. The results indicated that F127-GO-DOX had good biocompatibility and no obvious toxicity on the mice even under NIR laser irradiation.

## Conclusions

In this work, we mixed GO and Pluronic polymer F127 to prepare a supramolecular hybrid hydrogel F127-GO. Then its morphology and structure were confirmed by TEM, SEM, AFM, FTIR and XRD. Besides such advantages as good biocompatibility and simple preparation, the main advantages which are worth mentioning are the following: firstly, the supramolecular hybrid hydrogel has an effective loading content of DOX which can even reach 100%. *In vitro* drug release experiments indicate that the hydrogel system has a sustained-release characteristic which results from the erosion of the hydrogel matrix and the non-covalent interactions between DOX and the GO surface. Furthermore, the strong photothermal effect of GO significantly increased the treatment effect of the hydrogel system, especially under infrared laser irradiation whether *in vitro* or *in vivo*. All these properties make the supramolecular hydrogel a potential candidate for a sustained drug delivery system and combination treatment of chemotherapy and photothermal therapy.

## Conflicts of interest

There are no conflicts to declare.

## Supplementary Material
